# Expression of alpha smooth muscle actin decreases with ageing and increases upon lumen obstruction in mouse brain pericytes

**DOI:** 10.1007/s11357-024-01429-0

**Published:** 2024-11-26

**Authors:** Fanni Győri, Ádám Mészáros, Mónika Krecsmarik, Kinga Molnár, Cornel Balta, Anca Hermenean, Attila E. Farkas, István A. Krizbai, Imola Wilhelm

**Affiliations:** 1 Institute of Biophysics, HUN-REN Biological Research Centre, Szeged, Hungary; 2 Theoretical Medicine Doctoral School, University of Szeged, Szeged, Hungary; 3 Aurel Ardelean” Institute of Life Sciences, Vasile Goldiș Western University, Arad, Romania

**Keywords:** Ageing brain, Alpha smooth muscle actin (α-SMA), Ensheathing pericyte, Mesh pericyte, Microocclusion

## Abstract

Cerebral pericytes are mural cells covering brain microvessels, organized as ensheathing, mesh and thin-strand pericytes. These latter two, together called capillary pericytes, have low levels of alpha smooth muscle actin (α-SMA), regulating basal vascular tone and applying a slow influence on cerebral blood flow. Pericytes are subject to alterations in ageing which may be even more pronounced in age-related pathologies, including microinfarcts, which usually affect a large number of vessels in the ageing brain. We modelled this condition by injecting 10 µm-size microspheres into the circulation of mice resulting in the occlusion of capillaries covered by ensheathing and mesh pericytes. We observed that α-SMA and *Acta2*, the gene encoding it, as well as TGF-β1/*Tgfb1*, the major regulator of α-SMA, decreased during ageing in cerebral microvessels. In the vicinity of the microspheres stalled in the capillaries, expression of α-SMA increased significantly in both ensheathing and especially in mesh pericytes, both in young (2 to 3 months of age) and old (24 months of age) mice. On the other hand, γ-actin was detected in endothelial cells, but not in pericytes, and decreased in microvessels of microsphere-containing hemispheres. Altogether, our data show that obstruction of cerebral microvessels increases α-SMA expression in pericytes in both age groups, but this does not compensate for the lower expression of the contractile protein in old animals. Increased α-SMA expression may lead to constriction of the obstructed vessels probably aggravating flow heterogeneity in the aged brain.

## Introduction

The neurovascular unit (NVU) represents a close structural and functional relationship between cerebral microvascular cells (endothelial cells and pericytes) and neural cells (neurons and glia) having two main functions, regulation of cerebral blood flow (CBF) and the blood–brain barrier (BBB). Among cells of the NVU, pericytes are the least well-defined and most controversial cells. They are mural cells located in the duplication of the basement membrane of microvessels, coming in close contact with cerebral endothelial and glial cells. Although discovered almost 150 years ago, pericytes remain largely uncharacterized cells, heterogenic in their morphology and function, only defined by their perivascular localization [1].

Pericytes are multipotent cells, which form a phenotypic and probably functional continuum with vascular smooth muscle cells (VSMCs) to regulate key functions of the NVU [2]. According to their morphology and alpha smooth muscle actin (α-SMA) expression, as well as their calcium signalling characteristics and specificity for dye uptake, mural cells covering microvessels arising from penetrating arterioles are organized as ensheathing, mesh and thin-strand pericytes [3–5]. These cells are preceded upstream by circular VSMCs surrounding penetrating arterioles and often by precapillary sphincter cells at the transition between the penetrating arteriole and first-order capillary [6] and followed downstream by venular mesh pericytes and venule VSMCs on the post-capillary end of the vascular network.

Ensheathing pericytes are short cells in their length, having a protruding cell body, exhibiting a high vessel coverage and expressing detectable amounts of α-SMA. They have been also called transitional pericytes or pre-capillary VSMCs [7]. These cells usually cover the first four branches ramifying from the penetrating arteriole. On the other hand, mesh and thin-strand pericytes, together called capillary pericytes, have low levels of α-SMA that are usually detectable using special fixation techniques [8] and are downstream of the α-SMA-terminus, i.e. the halt of α-SMA staining, which is seen with conventional labelling methods [3, 9]. Mesh pericytes are longer than ensheathing pericytes, endowing microvessels of approximately 6 µm diameter with an intermediate coverage in the mouse brain, while thin-strand pericytes have long processes along the length of the smallest capillaries with small perpendicular processes partly embracing the vessel. Interestingly, NeuroTrace 500/525, a fluorescent Nissl stain used to label neuronal somata, is specifically taken up in vivo by capillary pericytes [5].

Considering their heterogeneity, it is not surprising that the role of pericytes in the regulation of CBF has been intensively debated and initially thought to be restricted to transitional/ensheathing cells [10–12]. Ensheathing pericytes encircling the vessel lumen were shown to control both vasodilation and vasoconstriction in the brain, first-order vessels dilating even before penetrating arterioles covered by VSMCs [10]. However, selective ablation of capillary pericytes led to vasodilation [13], while their optogenetic stimulation decreased lumen diameter [14]. These data indicated that capillary pericytes take part in the regulation of the basal vascular tone and the slow modulation of CBF.

Involved in several aspects of neurovascular functions, pericytes are subject to alterations in both normal and pathological ageing. Age-related morphological and functional changes of the NVU, including vessel rarefaction, micro-haemorrhages, increased BBB permeability, as well as diminished CBF and reduced neurovascular coupling are the consequence of cellular and molecular changes, which involve senescence and inflamm-ageing [15, 16]. Pericyte dysfunction and even pericyte loss are the main components of these alterations [17]; local pericyte loss results in focal capillary dilation that leads to increased flow heterogeneity [18]. Alterations in cell contractility may also contribute to age-related pericyte dysfunction. In this context, we aimed to understand changes in the expression and regulation of contractile proteins in pericytes during ageing.

Pericyte dysfunction is even more pronounced in ageing-related pathologies, including neurodegenerative and ischaemic conditions [19, 20]. Ischaemia was shown to induce pericyte contraction that could be followed by pericyte death in rigour, which may irreversibly constrict capillaries [10].

As parts of the cerebral small vessel disease spectrum, microinfarcts are one of the most common ischaemic lesions of the ageing brain, contributing significantly to cognitive decline and dementia [21, 22]. Microinfarcts affect a large number of vessels globally throughout the brain; thus, the best method to model this condition is injection of microspheres into the circulation [23]. Using this technique, regionally selective neuronal injury was observed [23]. However, changes in pericytes, especially in their expression of contractile proteins remain largely unknown in response to microinfarcts. Therefore, our second objective was to understand the changes in α-SMA expression in obstructed microvessels.

Finally, we hypothesized that other actin isoforms than α-SMA might be involved in the regulation of pericyte contractility.

In the present study, we observed that obstruction of cerebral microvessels increases α-SMA expression in pericytes in both young and old mice, but this does not compensate for the lower expression of the contractile protein in old animals. In addition, while searching for other contractile elements, we found that γ-actin is expressed in endothelial cells but not in pericytes.

## Materials and methods

### Animals

All mice were housed and treated in accordance with the Directive 2010/63/EU of the European Parliament on the protection of animals used for scientific purpose, and the protocols were approved by the institutional care and the Regional Animal Health and Food Control Station of Csongrád-Csanád County (permit numbers: XVI./767/2018 and XVI./2161/2024). Altogether, 26 young (2 to 3 months of age) and 21 old (24 months of age) BALB/c mice (Charles River Laboratories, Wilmington, MA, USA) were used in the study.

### Microvessel isolation

Young and old mice were anaesthetized with Avertin (a mixture of 2,2,2-tribromoethanol, 2-methyl-2-butanol and ethanol) and transcardially perfused with phosphate buffered saline (PBS, 0.1 M, pH = 7.4). Brains were taken out and the meninges were removed on Whatman paper. The brain tissues were homogenized in Dulbecco’s modified Eagle’s medium/Nutrient Mixture F-12 (DMEM/F-12; Thermo Fisher Scientific, Waltham, MA, USA), then digested with collagenase II (Merck-Sigma, St. Louis, MO, USA) and DNase (Merck-Sigma) for 30 min, at 37 °C, and 250 rpm shaking. Samples were diluted with DMEM/F12 and centrifuged at 1000 × g for 8 min at 4 °C. The pellets were resuspended in 20% bovine serum albumin (BSA) (VWR International, Radnor, PA, USA) and centrifuged at 1000 × g for 20 min at 4 °C. The pellets were suspended in DMEM/F12 and filtered through a 70-µm pore-size cell strainer (Corning, Corning, NY, USA) then centrifuged at 700 × g for 5 min at 4 °C. The pellets were resuspended in PBS, and the quality of the microvessels was checked under a microscope. Finally, the samples were centrifuged at 600 × g for 5 min at 4 °C. The pellets were suspended in TRI Reagent (Thermo Fisher Scientific; for RNA isolation) or radioimmunoprecipitation assay (RIPA) buffer (for western blot). For immunofluorescence, the microvessel fraction was fixed in 4% paraformaldehyde (PFA).

### RNA isolation and real-time polymerase chain reaction (qPCR)

Total RNA was isolated using Ambion RNAqueous®-Micro Kit (Thermo Fisher Scientific). For reverse transcription, the Maxima First Strand cDNA Synthesis Kit (Thermo Fisher Scientific) was used. Amplification was performed using the iTaqTM Universal SYBR Green Supermix (Bio-Rad, Hercules, CA, USA) on a Bio-Rad iQ5 instrument using mouse *Acta2* (fw: GAGCGTGGCTATTCCTTCGTG; rv: CAGTGGCCATCTCATTTTCAAAGT), *Tgfb1* (fw: CACCGGAGTTGTGCGGCAGT; rv: TGCCGCACGCAGCAGTTCTT) and *GAPDH* (fw*:* GGTCTTCCTCGAAGCACTT; rv: GTGAAGACGCCAGTAGACTC) primers.

### Western blot

Microvessel samples collected in RIPA buffer were centrifuged at 16,000 × g for 15 min at 4 °C. Supernatants were collected and protein concentration was determined by the bicinchoninic acid assay (Thermo Fisher Scientific). After addition of Laemmli buffer, the samples were incubated at 95 °C for 5 min. Samples were electrophoresed using standard denaturing SDS/PAGE and blotted on polyvinylidene difluoride membranes (0.2 µm pore size; Bio-Rad). After blocking with 3% BSA or 5% nonfat milk in Tris-buffered saline containing 0.1% Tween-20 (TBS-T), membranes were incubated with primary antibodies overnight at 4 °C, as follows: anti-α-SMA (Abcam, Cambridge, UK; cat. no. ab5694) in a dilution of 1:2000, anti-TGF-β1 (Santa Cruz Biotechnology, Dallas, TX, USA; cat. no. sc-130348) in a dilution of 1:500, anti-γ-actin (Abcam; cat. no. ab123034) in a dilution of 1:1000 and anti-β-actin (Merck-Sigma; cat. no. A5441) in a dilution of 1:10,000. Blots were washed in TBS-T three times for 10 min, incubated for 1 h in horseradish peroxidase (HRP)-conjugated anti-rabbit IgG or anti-mouse IgG secondary antibodies (Jackson Immunoresearch, Cambridgeshire, UK) diluted to 1:3000 in TBS-T and then washed again in TBS-T. Immunoreaction was visualized with the Clarity Chemiluminescence Substrate (Bio-Rad) in a ChemiDoc MP System (Bio-Rad). Densitometry analysis was performed with the IMAGE LAB Software, version 6.0.0 (Bio-Rad).

### Surgeries

#### Microocclusion model

Mice were anaesthetized via inhaled isoflurane 4% (v/v) in oxygen for induction and 1–2% (v/v) for maintenance, from a precision vaporizer (Open Circuit Isoflurane Tabletop System, Stoelting, Dublin, Ireland). The depth of anaesthesia was monitored by the toe-pinch test. After dissection of the connective tissue and retraction of the muscles, the right common carotid artery was carefully isolated from the tissue. The external carotid artery was temporarily ligated with surgical thread. A temporary loop was placed on the proximal section of the common carotid artery, and a sterile cotton ball was placed under the distal section to lift it. A 30G needle was inserted into the artery to inject 100 µl of PBS containing ~ 4000 Fluoresbrite® YG microspheres (10 µm; Polysciences, Warrington, PA, USA). In control animals, 100 µl of PBS was injected into the circulation. After the injection, bleeding was controlled using a hemostatic sponge and sterile cotton swabs, and the loops were removed to restore circulation. After 2, 4 or 24 h, mice were transcardially perfused with PBS, flowed by 100% methanol (MeOH) for actin stabilization, and finally 4% PFA. The brains were removed and post-fixed with PFA at 4 °C overnight. On the next day, the brains were placed into 30% sucrose solution in PBS and stored at 4 °C until sectioning.

For microvessel isolation, mice received ~ 250,000 Fluoresbrite® YG microspheres in the right internal carotid artery. After 4 h, microvessels were isolated as described previously separately from the two hemispheres and collected in RIPA buffer.

#### Microinjection and sample preparation

To block F-actin depolymerization in vivo, stereotaxic injections of jasplakinolide (10 µM in PBS-DMSO; Tocris, Bio-Techne; cat. no. 2792) combined with 0.05% Fluorescein-dextran (70 kDa; Merck-Sigma) to see injection tracks were performed unilaterally at four sites into the parietal cortex of mice. Injection was carried out using a glass micropipette (Drummond Scientific Company, Broomall, PA, USA) with a long narrow tip (~ 20 µm) pulled with a micropipette puller (Sutter Instrument, Novato, CA, USA). The micropipette was inserted into the brain through a burr hole of 1–1.5 mm diameter, and jasplakinolide was infused at a flow rate of 6 nl/s at depths of 200 µm, 300 µm, 400 µm, 500 µm and 600 µm (100 nl per injection location) with a programmable nanolitre injector (Nanoject III, Drummond Scientific Company). Two hours after intracerebral injection, in order to have a quick fixation to avoid actin depolymerization, brains were collected after decapitation, and 2 mm-thick pieces of tissue containing the injection sites were immediately cut out in the brain matrix soaked in ice-cold PBS. To preserve the antigenicity of actin [24], tissues were immersed in ice-cold Methacarn (MeOH-chloroform-glacial acetic acid 6:3:1) and kept at 4 °C for overnight. Methacarn was changed twice with 100% MeOH (for 2 h and then 3 h), followed by descending MeOH series (95%, 75%, 50%, 25%), each for two or more hours. Finally, the tissues were transferred to and stored in 0.1 M PBS until sectioning. Sections of 30 or 50 µm were cut on a cryostat (Leica CM1860) and air-dried on silane-coated glass microscope slides.

### Immunofluorescence, fluorescence microscopy and quantification of signals

#### Immunofluorescence staining

Isolated microvessels were placed onto adhesive microscope slides. Blocking and permeabilization were performed with PBS containing 3% BSA and 0.5% Triton X-100 (Merck-Sigma) for 1 h. Primary antibodies were added in the blocking solution in which microvessels were incubated overnight at 4 °C. The antibodies were the following: anti-CD13 (Bio-Techne, Minneapolis, MI, USA; cat. no. AF2335) in a dilution of 1:200 and collagen IV (Abcam; cat. no. ab6586) in a dilution of 1:100. After washing in PBS, microvessels were incubated with secondary antibodies diluted to 1:500 in PBS. Anti-goat Alexa Fluor 488 (Thermo Fisher Scientific; cat. no. A32814) and anti-rabbit Alexa Fluor 594 (Thermo Fisher Scientific; cat. no. A32754) were used.

Fixed brains containing microspheres were mounted onto a freezing microtome (Reichert-Jung, Leica, Wetzlar, Germany) and 30 µm-thick coronal sections were cut, which were stored in PBS containing 0.02% sodium azide. Antigen retrieval was performed by incubating the sections in 10 mM sodium citrate (pH = 6) for 10 min at 85 °C. All sections were permeabilized in 0.5% Triton X-100 for 30 min then blocked with 3% BSA in PBS containing 0.3% Triton X-100 for 1 h. Primary antibodies were diluted in PBS containing 3% BSA and 0.5% Triton X-100, and sections were incubated overnight at 4 °C on an orbital shaker. The following antibodies were used: anti-CD13 (Bio-Techne, Minneapolis, MI, USA; cat. no. AF2335) in a dilution of 1:200 and anti-α-SMA (Abcam; cat. no. ab5694) in a dilution of 1:100. Sections were extensively washed in PBS. Anti-goat Alexa Fluor 594 (Thermo Fisher Scientific; cat. no. A-11058) and anti-rabbit STAR RED (Abberior, Göttingen, Germany; cat. no. 2–0012-011–9) were applied as secondary antibodies in a dilution of 1:500 in PBS for 1 h at room temperature in the dark. Sections were washed, counterstained with a nuclear dye (Hoechst 33342; Merck-Sigma; dilution, 1:1000) for 10 min, washed again with PBS, rinsed in water and mounted in FluoroMount-G (SouthernBiotech, Birmingham, AL, USA).

For γ-actin staining, sections from brains that had been injected with jasplakinolide were used. The staining was performed as described above. As a primary antibody, anti-γ-actin (Abcam; cat. no. ab123034) and vWF (Abcam; cat. no. ab6994) were used in a dilution of 1:50, while the secondary antibodies were anti-mouse Alexa Fluor 647 (Thermo Fisher Scientific; cat. no. A32787) and anti-rabbit Alexa Fluor 488 (Thermo Fisher Scientific; cat. no. A32790) diluted to 1:500.

#### Fluorescence microscopy

Samples were analysed using a STEDYCON (Abberior Instruments, Göttingen, Germany) built on an Axio Observer Z1 inverted epifluorescence microscope (Zeiss, Oberkochen, Germany) equipped with an alpha Plan-Apochromat 100 × /1.46 oil immersion objective*.* Confocal Z-stack images for statistical analysis were captured at 100 × magnification using the same settings for each image.

#### Quantification of the signals

The percentage of α-SMA-positive microvessels blocked by microspheres was calculated by counting the total number of microspheres in 7–12 sections and the number of microspheres localized in α-SMA-positive vessels in the same sections. The ratio was calculated by dividing the microspheres found in α-SMA-positive vessels with the total number of microspheres.

The mean grey values of α-SMA signals were measured using the ImageJ software. The ROIs were selected manually based on the presence of the CD13 signal. The measurements of vessels with lodged microspheres (26 vessels from 2 young, 23 vessels from 2 old mice) were compared to control vessels (69 vessels from 3 young, 82 vessels from 3 old mice). Additionally, vessels had to meet the criterion of containing CD13-positive pericytes, which were categorized as being ensheathing, mesh or thin-strand based on the morphology. Ensheathing pericytes were recognized as elongated mural cells enwrapping the whole vessel, having a protruding cell body, while mesh pericytes were considered those perivascular cells which incompletely covered the endothelium, frequently localized at branching points.

### Statistical analysis

Values are expressed as mean ± SEM. Two-group comparisons were performed with Student’s *t*-test using the Excel 2016 Data Analysis plugin. Unless otherwise noted, multiple comparisons were evaluated using one-way analysis of variance (ANOVA), and post hoc comparisons were assessed by Tukey’s multiple comparisons test using GraphPad Prism 8. The significance level between groups was defined as *P* < 0.05.

## Results

**Fig. 1:**
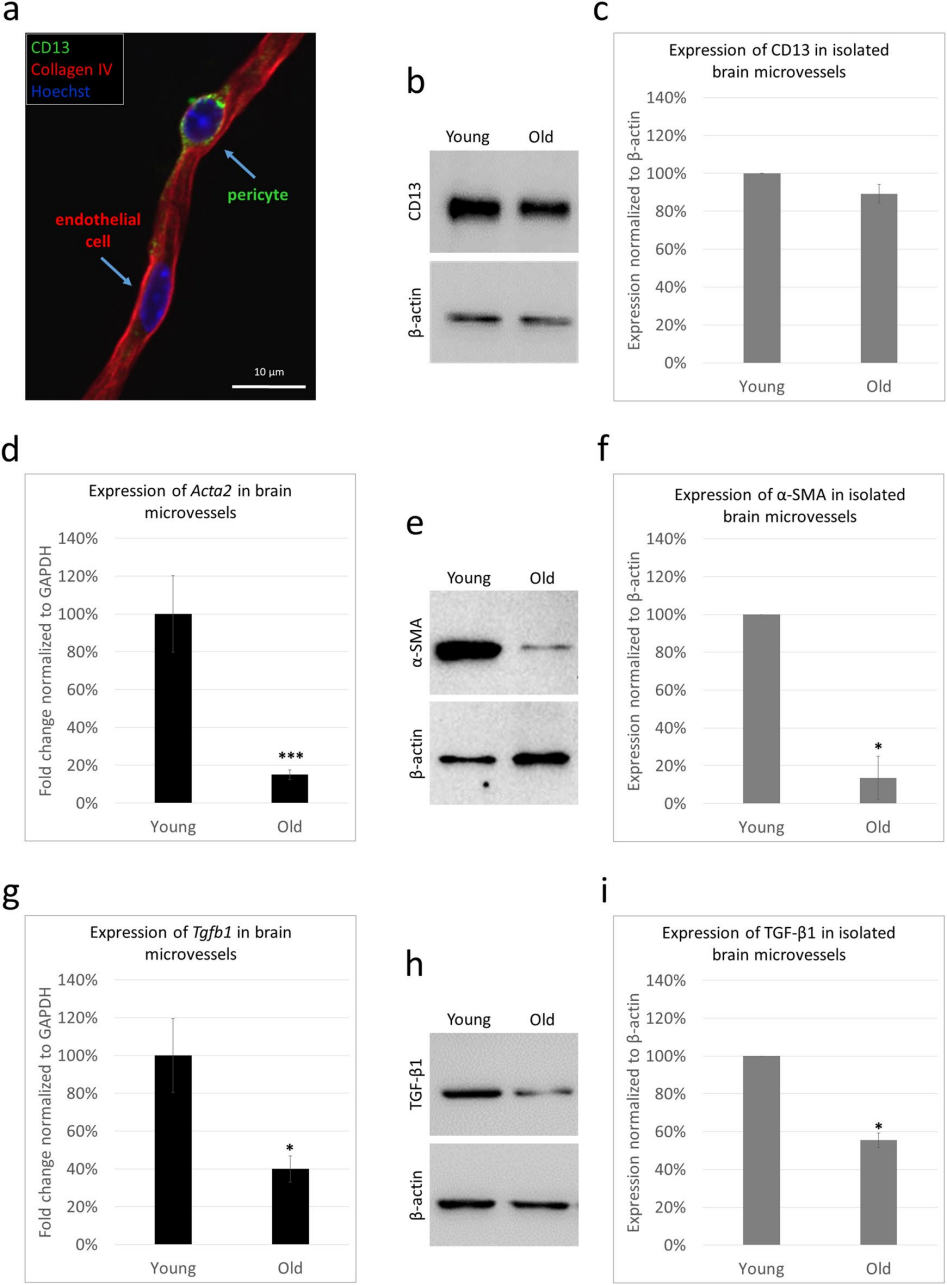
Expression of *Acta2*/α-SMA and *Tgfb1*/TGF-β1 in brain microvessels of young and old mice. **a** Representative immunofluorescence micrograph (maximum intensity projection) showing an isolated brain microvessel. **b** Representative western blot showing the expression of CD13 in brain microvessels of young and old mice (β-actin was used as a loading control). **c** Quantitative analysis of CD13 levels compared to β-actin expression, as assessed by western blot. Graphs represent the average ± SEM (*N* = 2). **d** Gene expression of *Acta2* in microvessels isolated from the brains of young (2 to 3 months of age) and old (24 months of age) BALB/c mice. Graphs represent the fold change (normalized to *GAPDH*), average ± SEM (*N* = 2 independent experiments, each performed in triplicate). ***P ≤ 0.001 (Student’s *t*-test). **e** Representative western blot showing the expression of α-SMA in brain microvessels of young and old mice (β-actin was used as a loading control). **f** Quantitative analysis of α-SMA levels compared to β-actin expression, as assessed by western blot. Graphs represent the average ± SEM (*N* = 2). *P ≤ 0.05 (Student’s *t*-test). **g** Gene expression of *Tgfb1* in microvessels isolated from the brains of young (2 to 3 months of age) and old (24 months of age) BALB/c mice. Graphs represent the fold change (normalized to *GAPDH*), average ± SEM (*N* = 2 independent experiments, each performed in triplicate). ***P ≤ 0.001 (Student’s *t*-test). **h** Representative western blot showing the expression of TGB-β1 in brain microvessels of young and old mice (β-actin was used as a loading control). **i** Quantitative analysis of TGB-β1 levels compared to β-actin expression, as assessed by western blot. Graphs represent the average ± SEM (*N* = 2). *P ≤ 0.05 (Student’s *t*-test)

To investigate contractile protein expression in the cerebral microvessels of young and old mice, we first isolated small vessels from the brains of young (2 to 3 months of age) and old (24 months of age) BALB/c mice. Isolated microvessels contained endothelial cells and pericytes (Fig. 1a).

Comparing microvessels of young and old mice, we observed that the amount of CD13 (aminopeptidase N) protein did not change significantly with ageing (Fig. 1b, c). On the other hand, expression of *Acta2*/α-SMA decreased drastically both on the mRNA and protein levels in the microvessels of old mice in comparison to microvessels isolated from the brains of young animals (Fig. 1d–f). Since the decrease in the amount of α-SMA was much more pronounced than that of CD13, this suggests that no change in pericyte number but rather that of protein expression is responsible for the observed phenomenon.

In both VSMCs and pericytes, one of the main regulators of α-SMA expression is TGF-β1 [25, 26]. Therefore, we next analysed the expression of *Tgfb1*/TGF-β1 and detected significantly lower gene and protein expression levels in the 2-year-old mice than in the young (Fig. 1g–i). This is in line with the decreased expression of *Acta2*/α-SMA, most probably contributing to this latter.

**Fig. 2:**
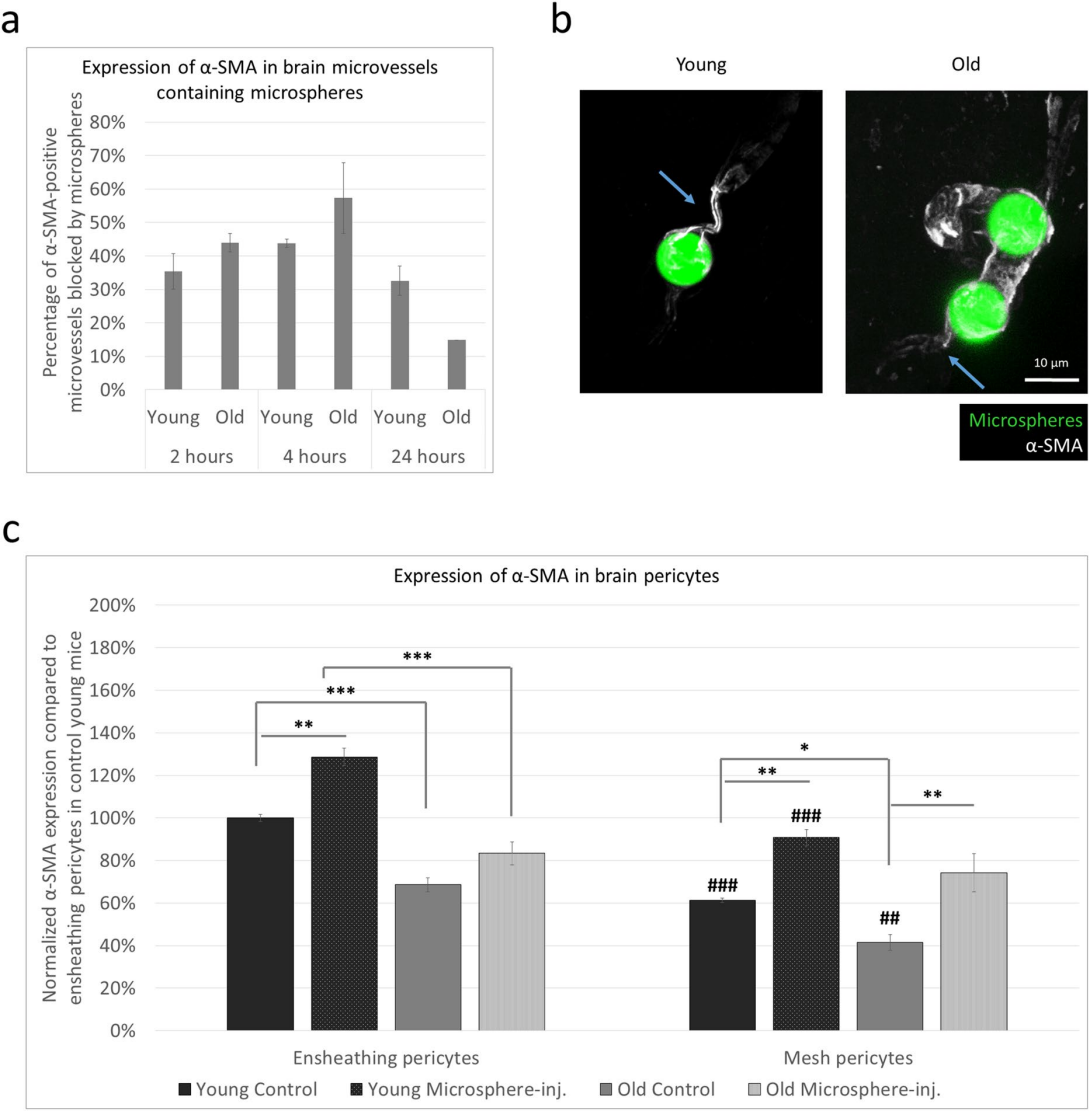
Expression of α-SMA in brain microvessels containing microspheres in young and old mice. **a** Time-dependent expression of α-SMA in microvessels blocked by microspheres, measured by immunofluorescence intensity in young (2 to 3 months of age) and old (24 months of age) BALB/c mice. The graph represents the average ± SEM (*n* = 7–12 brain sections per animal, *N* = 2 animals per group). No significant difference among the groups (two-way ANOVA and Sidak’s multiple comparisons test). **b** Representative immunofluorescence micrographs (maximum intensity projection) showing vasoconstriction in the vicinity of the microspheres (indicated by arrows). **c** Expression of α-SMA in ensheathing and mesh pericytes measured by immunofluorescence intensity in brain sections of young (2 to 3 months of age) and old (24 months of age) BALB/c mice at 4 h after injection of the microspheres. The graph represents the average ± SEM (*n* = 5–10 cells per animal, *N* = 2 animals per group). *P ≤ 0.05, **P ≤ 0.01, ***P ≤ 0.001; ##*P* ≤ 0.01, ###*P* ≤ 0.001 compared to ensheathing pericytes of the same group (ANOVA and Tukey’s multiple comparisons test). Microsphere-inj, microsphere-injected

As cerebral microinfarcts are common in ageing, we next aimed at understanding how the contractility of mural cells might influence the outcome of vessel obstruction. We modelled this condition by delivering fluorescent microspheres of 10 µm diameter into the brain vessels of mice via injection into the internal carotid artery. In accordance with previous results [23], we observed the highest number of microspheres blocked in the neocortex (in young mice, 33.43%, while in old mice, 32.47% of the total number), followed by the thalamus (15.01% and 13.90%, respectively) and the hippocampus (8.93% in young and 11.63% in old animals). Although not significantly higher than at 2 or 24 h, the number of α-SMA-positive microsphere-containing vessels was the highest 4 h after injection of the beads, both in young and old animals (Fig. 2a). Therefore, we performed all the subsequent experiments at this time point and focused on evaluating the cortical areas where the majority of the microspheres became lodged. Using immunofluorescence staining of α-SMA in brain sections, we observed a tendency of vasoconstriction in the vessels encompassing the microspheres, although a substantial narrowing of the lumen of blood vessels was a rare event, especially in old animals (Fig. 2b).

**Fig. 3:**
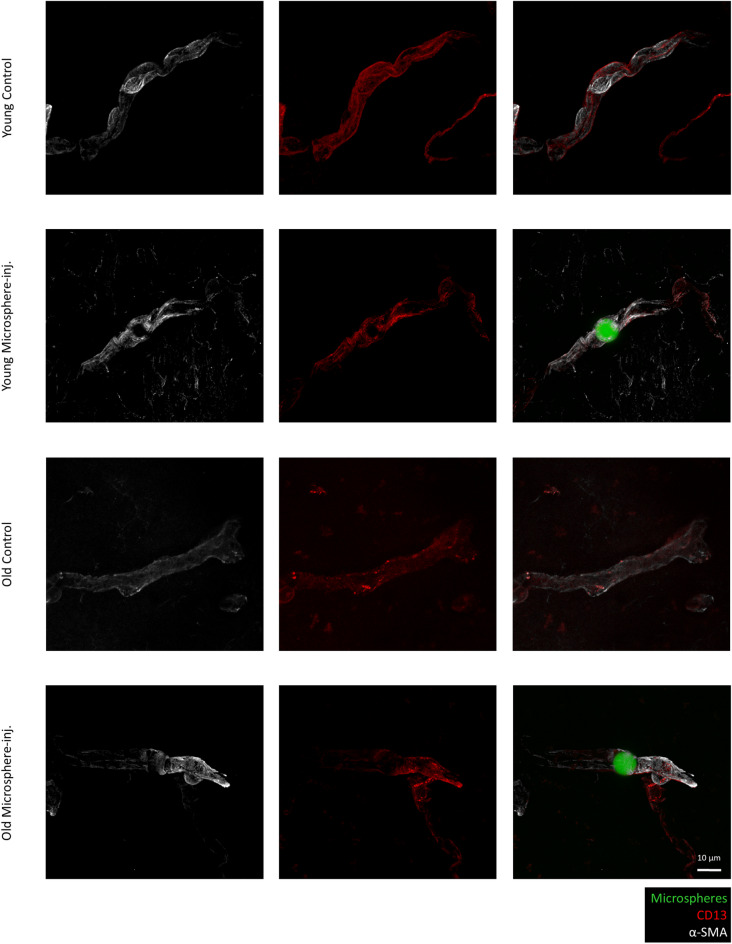
Expression of α-SMA and CD13 in ensheathing pericytes of brain microvessels containing microspheres. Representative immunofluorescence micrographs (maximum intensity projections) showing α-SMA and CD13 expression in brain sections of young (2 to 3 months of age) and old (24 months of age) BALB/c mice 4 h after the injection of the microspheres. Microsphere-inj, microsphere-injected

**Fig. 4:**
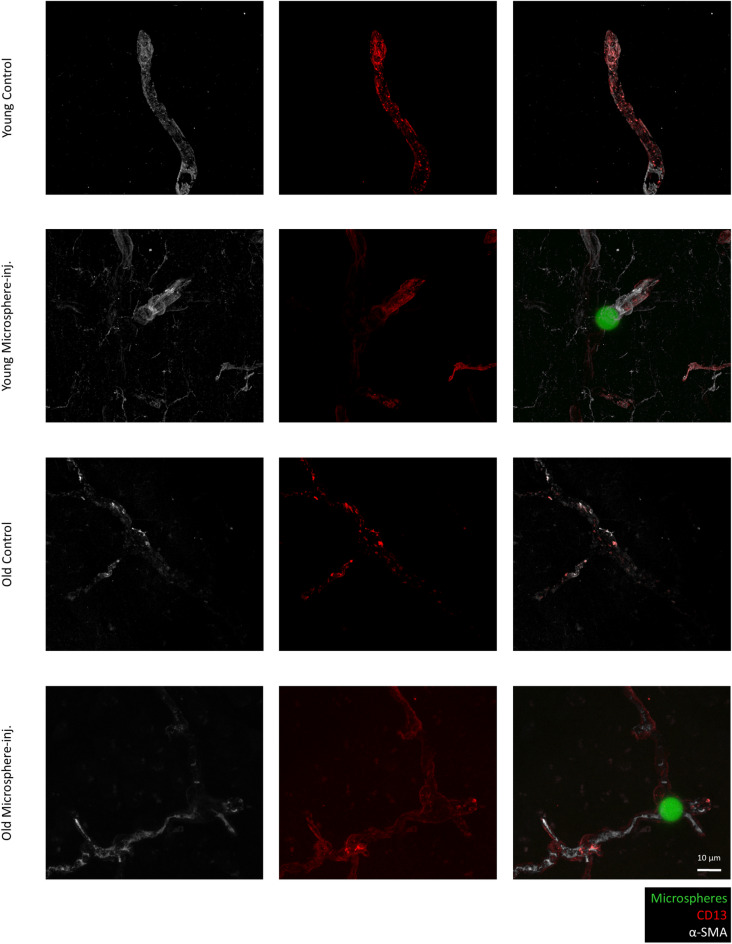
Expression of α-SMA and CD13 in mesh pericytes of brain microvessels containing microspheres. Representative immunofluorescence micrographs (maximum intensity projections) showing α-SMA and CD13 expression in brain sections of young (2 to 3 months of age) and old (24 months of age) BALB/c mice 4 h after the injection of the microspheres. Microsphere-inj, microsphere-injected

We quantified and compared α-SMA expression intensity in the microbead-containing and control hemispheres of young and old mice. As expected, in both age groups, ensheathing pericytes were richer in α-SMA than mesh pericytes. Decreased levels of α-SMA were detected in both ensheathing and mesh pericytes, respectively, in old animals in comparison to their young counterparts (Fig. 2c). In young mice, expression of α-SMA increased significantly in both ensheathing and mesh pericytes in response to the presence of the microspheres (Figs. 2c, 3 and 4). In ensheathing pericytes of old animals, there was only a tendency of higher α-SMA expression in microbead-containing vessels versus control vessels (Figs. 2c and 3), while in mesh pericytes of 24-month-old animals, α-SMA increased significantly surrounding microbead-containing vessels, approximately reaching the level of that measured in young control vessels (Figs. 2c and 4). Increased α-SMA expression was predominantly observed on one side of the microsphere; however, due to the thin sections used, we were unable to determine whether this occurred in the upstream or downstream direction. Changes in thin-strand pericytes are not shown since expression of α-SMA was very low in these cells and only detectable in young animals. Microspheres usually stalled in lower order vessels and were very rarely observed in capillaries covered by thin-strand pericytes.

**Fig. 5:**
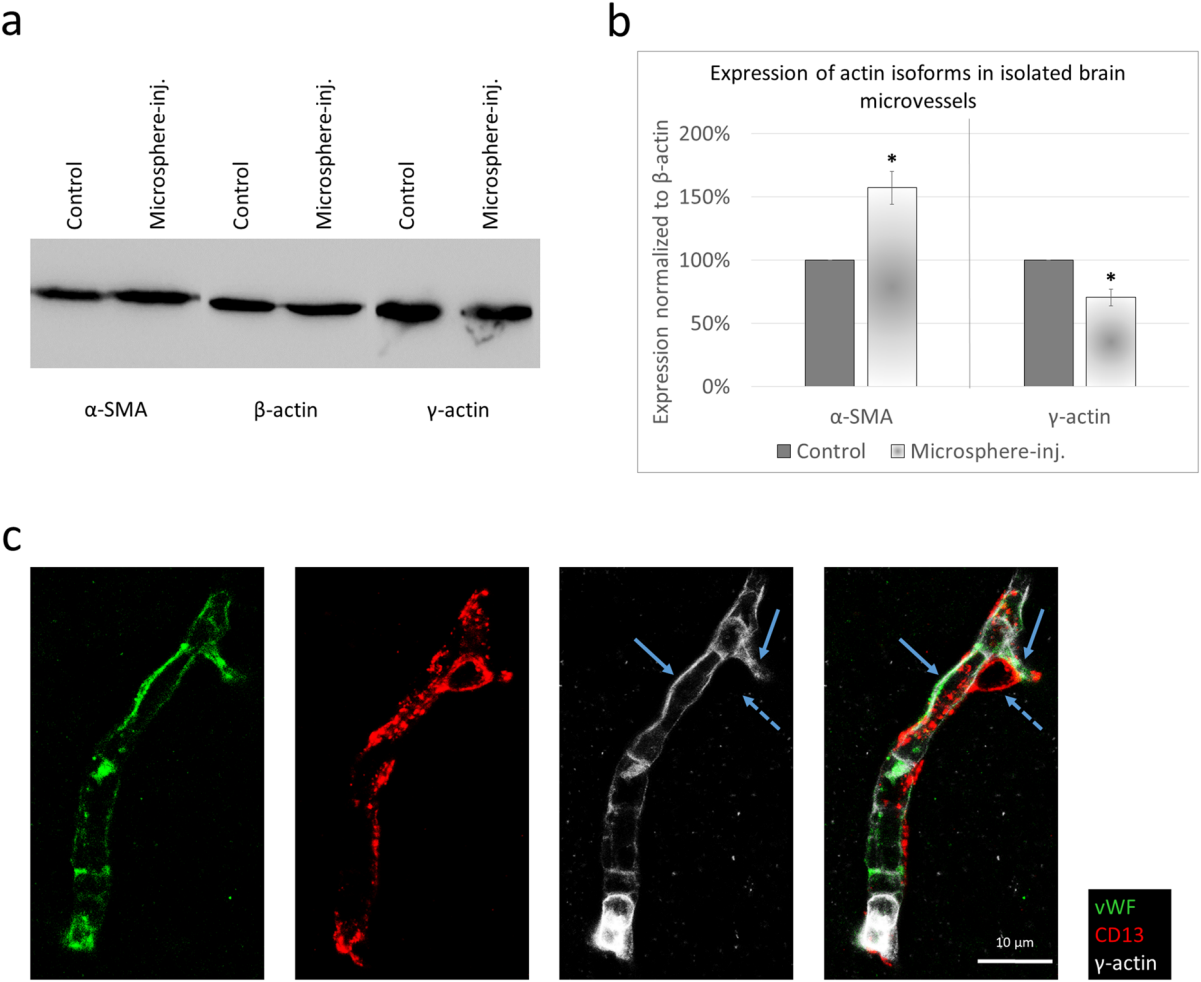
Expression of actin isoforms in brain microvessels. **a** Representative western blot showing the expression of α-SMA, β- and γ-actin in brain microvessels isolated from young mice injected with 10 µm-diameter microspheres. **b** Quantitative analysis of α-SMA and γ-actin levels compared to β-actin expression, as assessed by western blot. Graphs represent the average ± SEM (*N* = 2). *P ≤ 0.05 (Student’s *t*-test). **c** Representative immunofluorescence micrographs (maximum intensity projections) showing expression of γ-actin in brain sections of young BALB/c mice. Arrows denote endothelial localization of γ-actin. The dashed arrow indicates absence of γ-actin from pericytes

The contractility of pericytes and their involvement in the regulation of CBF has long been debated mainly due to the difficulties in the detection of α-SMA in thin-strand pericytes [27]. Contribution of other actin isoforms has been suggested, like γ-actin; however, recently, expression of γ-actin was detected only in pericytes of lower order vessels [8]. We wanted to acquire a more nuanced understanding of γ-actin expression in brain capillaries and to appreciate whether it shows similar changes as α-SMA in response to lumen obstruction. The same 10 µm-size microbeads were injected unilaterally, but in higher numbers to ensure the majority of microvessels are affected. As detected by western blot, α-SMA increased significantly. On the contrary, the expression of γ-actin decreased in microvessels isolated from the microsphere-containing hemispheres (Fig. 5a, b). Finally, we wanted to determine which vascular cells express γ-actin. By performing immunofluorescence staining in isolated microvessels, γ-actin was clearly detected in von Willebrand factor (vWF)-positive endothelial cells but was absent in CD13-positive pericytes (Fig. 5c), suggesting a difference between endothelial cells and pericytes in the expression of actin isoforms both in basal and injured conditions.

Changes in the expression contractile proteins in brain microvascular cells in response to ageing and microocclusion are summarized in Table 1.

**Table 1:** Changes in the expression contractile proteins in brain microvascular cells in response to ageing and microocclusion

Cerebrovascular cell type	Contractile protein	Ageing	Microocclusion
Pericyte (mesh, ensheathing)	α-SMA	↓↓↓	↑↑
Microvascular endothelial	γ-actin	?	↓

## Discussion

Proper neuronal function relies on a tightly regulated microcirculation that meets the high and continuous metabolic demands of the brain. VSMCs, precapillary sphincters and ensheathing pericytes take a robust activity in regulating vasoconstriction and vasodilation, while capillary pericytes rather participate in the modulation of the effect.

Ageing and ageing-related diseases alter the structure of the cerebral microvasculature, which exacerbates BBB leakage and leads to impaired CBF, as well as a decrease in neurovascular coupling [28–30]. However, the involvement of pericytes in these processes is far from being completely elucidated.

Here we observed that α-SMA and *Acta2*, the gene encoding it, decrease during ageing in microvessels isolated from mouse brains. Since CD13 did not show a similar change, we conclude that loss of α-SMA is due to a decreased α-SMA content and not a reduction in the density of the pericytes. Moreover, we have also detected a significant drop in vascular α-SMA expression in brain sections, which affected both ensheathing and mesh pericytes.

Previous studies on ageing-related changes in pericytic α-SMA expression and pericyte number are uncertain. In human post-mortem brains, a reduction in pericyte number was observed in the frontal cortex in normal ageing but not in vascular dementias and Alzheimer’s disease (AD) [31], while other studies described pericyte degeneration in AD [32] and in cognitive impairment [33]. Moreover, a decrease in the α-SMA content of VSMCs was observed in ageing and especially in AD [34, 35].

In mouse models of ageing, pericyte vulnerability and decreased remodelling were shown to result in BBB opening or deficits in CBF [18, 36, 37]. Recently, ageing was proven to decrease vascular responsivity, especially the relaxation ability of precapillary sphincters and of ensheathing pericytes in the mouse brain. This was accompanied by loss of processes, but not of the number or the α-SMA content of mural cells [38].

Although these results are somewhat controversial, several of them point to the reduction in the amount of α-SMA in mural cells, expressly in those with higher contractile protein content, *i.e.* VSMCs and ensheathing pericytes.

In our study, ageing-dependent decline in α-SMA expression of pericytes is further supported by the decreased expression of TGF-β1, which is the main stimulator of α-SMA transcription [39]. As previously shown, TGF-β1 induces growth arrest and a contractile phenotype in pericytes through Myf-5 and Smad2-mediated signalling [40]. Among cells of the BBB, both endothelial cells and pericytes, as well as astrocytes secrete TGF-β1 [41–43], having diverse effects on endothelial cells [42–45] and pericytes. As a limitation of our study, we have not tested which cell types downregulate TGF-β1 expression in the brain microvessels of old mice. In addition, it has to be noted that TGF-β signalling has diverse effects in cerebral ageing [46].

Ageing is a risk factor for brain ischaemic lesions as well, among which microinfarcts are very common. These are microscopic lesions with a wide distribution in the brain, affecting a large number of vessels [47]. Brain microinfarcts are often correlated with small vessel disease [48] and associated with cognitive dysfunction [49]. In our study, we aimed at understanding the involvement of pericyte contractility in the occlusion of cerebral microvessels. In order to target the capillary network, we used microspheres of 10 µm diameter, while previously 40–70 µm-sized cholesterol crystals or 20 µm-large microspheres had been injected into the circulation of mice to block penetrating arteries or arterioles, with the aim of understanding consequent neuronal damage [23, 50]. Using 10 µm microbeads, we primarily occluded capillaries covered by ensheathing and mesh pericytes, while higher order capillaries had been previously targeted using 4 µm-diameter microspheres [51].

We observed that pericytes reacted to vessel occlusion with increased expression of α-SMA, occasionally leading to vasoconstriction in both young and old mice. This is reminiscent of previous results indicating pericyte contraction and consequent capillary constriction in response to global cerebral ischaemia induced by middle cerebral artery occlusion [52]. This reaction might probably help in isolating the damaged microvessels, limiting the consequences of BBB breakdown [53]. However, vessel constriction may outlast perfusion blockage, and clamping pericytes might restrict reperfusion [20] and possibly die in rigour [10]. Alternatively, pericytes might possibly also facilitate recanalization or vessel pruning [51]. As a limitation of the present work, we have not assessed the direct effect of microocclusions on pericytes in vivo.

Single microocclusions presumably do not cause severe damage in the brain; however, the severity of an isolated microstroke largely depends on the local vascular topology [54]. In addition, accumulating microstrokes may contribute to a progressive loss of open capillaries, which might have an even worse outcome in the ageing brain already affected by microvascular rarefaction [55].

Out of other contractile elements, we investigated γ-actin, which was suggested to participate in the contractility of VSMCs [56]. Among the six actin isoforms, β- and γ-actin are the two which are non-muscle or cytoplasmic actin isoforms [57]. They differ by only four amino acids and are expressed in both muscle and non-muscle cells [58]. Expression of γ-actin has been detected in both endothelial cells [59] and pericytes [60]; however, expression of this actin isoform in pericytes of the central nervous system has been debated [8, 60]. Our results clearly indicated the presence of γ-actin in microvascular endothelial cells of the brain but not in pericytes. Expression of γ-actin did not increase but slightly decreased in response to lumen obstruction.

Taken together, pericyte dysfunction contributes significantly to ageing-associated cerebrovascular changes. Pericytes actively compensate for the dysfunction by extending long processes to cover endothelial cells [7]; however, pericyte remodelling is slower in the aged brain [18]. Changes in the expression of contractile proteins (including ageing-induced reduction in α-SMA content and increased expression in response to microstrokes) might also be compensatory mechanisms; however, further studies are needed to clarify this question. We have also observed that obstruction of cerebral microvessels increases α-SMA expression in pericytes in both age groups, but this does not compensate for the difference in the basal expression. Increased α-SMA expression may lead to constriction of the obstructed vessels, which probably aggravates flow heterogeneity and contributes to impaired capillary blood flow in the aged brain. In addition, we have shown that γ-actin is expressed more in endothelial cells than in pericytes; therefore, it is presumably not involved in the response of pericytes to ageing and microinfarcts.
